# Disclosure of sperm donation: a comparison between solo mother and two-parent families with identifiable donors

**DOI:** 10.1016/j.rbmo.2016.08.004

**Published:** 2016-11

**Authors:** Tabitha Freeman, Sophie Zadeh, Venessa Smith, Susan Golombok

**Affiliations:** a Centre for Family Research, University of Cambridge, Free School Lane, Cambridge, CB2 3RF, UK; b The London Women's Clinic, 113-115 Harley Street, London, W1G 6AP, UK

**Keywords:** disclosure, donor insemination, identifiable donors, single mothers by choice, solo mothers

## Abstract

Disclosure of donor conception to children was compared between solo mother and two-parent families with children aged 4–8 years conceived since the removal of donor anonymity in the UK. Semi-structured interviews were conducted with 31 heterosexual solo mothers and 47 heterosexual mothers with partners to investigate their decisions and experiences about identifiable donation and disclosure to their children. No significant difference was found in the proportion of mothers in each family type who had told their children about their donor conception (solo mothers 54.8%; partnered mothers 36.2%). Of those who had not told, a significantly higher proportion of solo mothers than partnered mothers intended to disclose (*P* < 0.05). Partnered mothers were more likely than solo mothers to feel neutral, ambivalent or negative about having used an identifiable donor (*P* < 0.05), and were less likely to consider children's knowledge of their genetic origins as extremely important (*P* < 0.05). These findings are relevant to provision of counselling services as it cannot be assumed that parents will tell their children about their origins or their entitlement to request the identity of their donor at the age of 18 years. Further qualitative research would increase understanding of solo mothers' attitudes towards disclosure.

## Introduction

The landscape of sperm donation in the UK has changed significantly over the past decade. One of the most fundamental transitions has been the introduction of identifiable sperm donation, which means that children conceived using sperm donated from 1 April 2005 onwards will be able to access identifying information about their sperm donor on reaching 18 years of age. Furthermore, in the UK Human Fertilisation and Embryology Act (1990, as amended 2008), an original clause requiring clinics to consider the child's ‘need for a father’ in the decision to offer fertility treatment was replaced with a requirement to consider the child's need for ‘supportive parenting’. Coupled with the introduction of intracytoplasmic sperm injection, which reduced the number of heterosexual couples requiring sperm donation, this legislative change has meant that single women now form a substantial and growing proportion of donor sperm recipients at UK clinics. The latest figures report non-partnered women comprising 15% of those undergoing fertility treatments with donated gametes in 2013 (Human Fertilisation and Embryology Authority, 2014), with those who have children being variously described as ‘solo mothers’, ‘single mothers by choice’ and ‘choice mothers’ (Bock, 2000, Graham, 2014, Hertz, 2006). These policy transitions have been accompanied by an increased cultural openness about donor conception, marked by a tidal change in public attitudes towards parental disclosure. Previous professional advice was for parents not to tell anyone, least of all their children, about their use of sperm donation; however, now the general consensus is that parental openness about donor conception, ideally in early childhood, is in the best interests of the child (Daniels, Taylor, 1993, Freeman, 2015, Nuffield Council on Bioethics, 2013).

Historically, rates of disclosure in families headed by heterosexual couple families have been very low, with most parents deciding against telling their children about their donor origins (Brewaeys et al, 1997, Golombok et al, 2002, Gottlieb et al, 2000, Nachtigall et al, 1998). Nevertheless, studies of anonymous sperm donation show that mothers' intentions to disclose are significantly higher in solo mother than in heterosexual couple families (Klock et al, 1996, Murray, Golombok, 2005), presumed to result from the need to explain the absence of a father (Brewaeys, 2010). A body of empirical evidence, however, is not yet available to support the claim that solo mothers actually disclose to their children at an early age or that they do so owing to the absence of a father in the home. This is partly because studies of disclosure in donor conception families have largely focused on heterosexual and lesbian couples (Brewaeys, 2010, Indekeu et al, 2013). Moreover, the few studies that have investigated disclosure decision-making in solo mother families have, by and large, reported mothers' intentions to tell when their children were in infancy or not yet conceived. Although more recent research suggests that the large majority of solo mothers have either disclosed or plan to do so (Landau and Weissenberg, 2010), longitudinal studies with heterosexual couple families reveal that intentions are not necessarily borne out in practice, and that the disclosure process can become increasingly difficult and, in some cases less likely, the older children become (Blake et al, 2010, Golombok et al, 2002, Readings et al, 2011). The level of agreement between parents within heterosexual couple families may also affect the realization of disclosure intentions (Daniels et al., 2009), a factor that is not relevant to solo mothers.

The introduction of identifiable donation adds another level of complexity to understanding differential disclosure patterns between solo mother and two-parent families, as its impact on disclosure rates is not yet known. Although there is some evidence that parents of children born through gamete donation have become more favourable towards disclosure (Golombok et al, 2011, Scheib et al, 2003) and identifiable donation (Scheib et al., 2000), it is not yet clear if and how the use of identifiable donors has shaped these trends. Some research does not support a link between the use of identifiable donors and increased rates of disclosure or intentions to disclose (Araya et al, 2011, Baetens et al, 2000, Gottlieb et al, 2000, Greenfeld, Klock, 2004, Lalos et al, 2007, Laruelle et al, 2011), whereas other studies have found a positive association (Brewaeys et al, 2005, Crawshaw, 2008, Godman et al, 2006, Greenfeld et al, 1998), including several reporting a general trend towards increased parental openness in recent years (Isaksson et al, 2012, Rosholm et al, 2010, Soderstrom-Anttila et al, 2010). Such trends may be the result of greater information being available for parents to share with their children. A lack of such information was a reason for non-disclosure previously identified in research on heterosexual couples (Daniels et al., 1995).

In Sweden, where donor anonymity was removed in 1985, a high proportion of parents intend to disclose the use of donor conception to their children, whereas a much smaller proportion actually seem to do so (Isaksson et al., 2012); it has been shown that sharing information about donor conception is complex and sometimes difficult, and requires the child to be an active participant in the process (Isaksson et al., 2016). Again, the conclusions about increased parental openness drawn from studies of families formed using identifiable donors tend to reflect high rates of parents' intentions to disclose. Further follow-up studies are required to ascertain if this is realised in increased levels of parental disclosure in practice. Moreover, there is a tendency to pool together findings relating to egg donation and sperm donation, despite these different forms of gamete donation raising qualitatively different issues for parents and children (Freeman, 2015) and disclosure rates seeming to be higher in egg donation families (Blake et al., 2013). Furthermore, the conclusions about parental openness drawn from studies of families formed using identifiable donors have been extrapolated from studies of couples.

As the distinction between intended and actual disclosure indicates, disclosure is a complex process that benefits from close empirical scrutiny. Recent studies have begun to focus attention on when, what and how children are told about their conception and what they understand (Blake et al, 2010, Daniels et al, 2009, Nachtigall et al, 1997, Shehab et al, 2008, Tallandini et al, 2016). It has been suggested that using a ‘family-building’ rather than ‘child-conception’ narrative may be most appropriate for the disclosure of donor information (Daniels and Thorn, 2001). In a study of parents' communication styles, MacDougall et al. (2007) found that some parents waited until what they felt was the ‘right time’ to tell their child about their donor conception whereas others used a ‘seed planting’ approach so that their child would have always known. Other research has shown a positive association between attendance at support group workshops and feelings of confidence about how and when to share this information (Crawshaw and Montuschi, 2013).

Investigations of the disclosure process have revealed high levels of ‘partial disclosure’, where children are told that they were conceived at a clinic without being informed about the use of donated gametes (Readings et al., 2011). The effect of a child's age at first disclosure on their response to discovering their donor origins has also been highlighted (Jadva et al., 2009). If told at a young age, children's responses to learning about their donor origins tend to be neutral or positive (Blake et al, 2010, Blake et al, 2014). Young children may express little interest in the donor or some curiosity in knowing more about this person (Lindblad et al, 2000, Lycett et al, 2005, Rumball, Adair, 1999, Scheib, Ruby, 2008, Snowden, 1990, Vanfraussen et al, 2003). As yet, little is known about how children's responses to disclosure might be affected by identifiable donation. A study by Scheib et al. (2005) of 29 adolescents with identifiable sperm donors in lesbian couple, heterosexual couple and solo mother families who learned of their origins at an early age found that most were comfortable with the nature of their conception and the majority (86%) reported being at least moderately likely to seek contact with their donor. Other studies, however, suggest greater variability. For example, children may decide against finding out about their donor to protect the feelings of their birth parents, and some may want non-identifying rather than identifying information (Vanfraussen et al, 2001, Vanfraussen et al, 2003). Again, family type may play a fundamental role in shaping a child's response to disclosure and having an open-identity donor (Beeson et al, 2011, Hertz, Mattes, 2013). As no father is present in solo mother families, children may be particularly interested in knowing the identity of their donor.

The aim of the present study was to obtain systematic data on the disclosure of donor conception to children born as a result of sperm donation after the removal of donor anonymity in the UK. The study focused on parents' disclosure practices following this change in legislation and on whether there was evidence for the presumption of greater openness in solo mother than in two-parent families created using identifiable sperm donors.

## Materials and methods

### Participants

The sample comprised 31 heterosexual solo mothers and a comparison group of 47 heterosexual married or cohabiting mothers, all of whom had a child aged 4–8 years conceived using an identifiable sperm donor. Participants were recruited through a fertility clinic with one of the largest and longest established programmes providing donor insemination to single women in the UK. A random sample of single mothers with a child in the required age range was invited to participate. The inclusion criteria were as follows: single at the time of treatment, no cohabiting partner since the child's birth, no non-cohabiting relationship of more than 6 months since the child's birth and no use of egg donation in addition to sperm donation. Where mothers had more than one child in the required age range, the eldest eligible child was included in the study, and where the mother had twins, one was randomly selected to take part. A sample of partnered mothers was matched overall to the solo mothers according to child's age and gender, i.e. so that the groups were closely comparable in mean age of the children and the proportion of boys and girls. Partnered mothers were required to still be living with the child's father. Participation rates of 85% and 63% were obtained for the solo and partnered mothers, respectively.

As shown in Table 1, no difference was found between family types in the age or gender of the target child. A significant difference, however, was found in mother's age (F[1, 76] = 33.05, *P* < 0.001), reflecting the older age of the solo mothers. A difference was also found between family types in the number of siblings in the family (χ^2^[2] = 8.27, *P* < 0.05), with fewer siblings in solo mother families. No difference was found between the solo mother and two-parent families in either the mothers' working status, perceived financial difficulties or the mothers' educational qualifications, with more than one-half of the mothers in both family types having a university degree. Most solo mothers (29 [93.5%]) had never been married. The two (6.5%) solo mothers who had been married had divorced before their fertility treatment. Most (43 [91.5%]) partnered mothers were married and four (8.5%) were cohabiting. Using the Office of National Statistics (2011) classification, all mothers identified their ethnic group as white, with the exception of three who identified as black (two solo mothers and one partnered mother) and two as ‘mixed’ white and Asian (one solo mother and one partnered mother).

### Procedure

Mothers were given an audio-recorded interview lasting between 1 and 2.5 h at home by a trained researcher. Findings from a section of the interview that focused on the mother–child relationship and the psychological adjustment and experiences of the child are reported elsewhere (Golombok et al., 2016). All mothers gave written informed consent to participate in the study. Ethical approval was granted by the University of Cambridge Psychology Research Ethics Committee on 15 October 2015 (reference number: PRE.2015.089).

### Measures

The semi-structured interview was designed to assess parents' experiences of disclosure and has been validated in previous studies of donor conception families with children of the same age (Golombok et al, 2011, Readings et al, 2011). Detailed accounts were obtained from the mother about her experience of donor insemination using an identifiable donor and of telling, or not telling, her child about their conception. A flexible style of questioning was used to elicit sufficient information for the variables below to be rated by the researcher using a standardized coding scheme. Therefore, ratings were made by the researcher using in-depth information obtained from the mother rather than by the mother herself.

#### The disclosure decision

**Disclosure status** was rated as disclosed (child told that their conception was by donor insemination), partially disclosed (child told their conception required medical intervention without mentioning donor sperm or the donor), or not disclosed (no discussion of donor insemination or medical intervention); **disclosure intention** was rated as disclosed, plans to tell, uncertain, or plans not to tell. **Difficulty in disclosure decision-making** was rated as none/minor difficulties, or definite difficulties.

#### The disclosure process

**Method of disclosure** was rated as by book, by conversation or by story-telling; **initiator of discussions about donor insemination** was rated as predominantly mother, mother and child equally, or predominantly child; **frequency of discussions** was rated as sometimes (at least once every 3 months), occasionally (every 3–6 months), or rarely/never (less than every 6 months); **mothers' feelings about discussions** was rated as no problems, mildly uncomfortable/distressed, or uncomfortable/distressed; **child's feelings about being donor conceived** was rated as positive, neutral/mixed, mother unsure of child's feelings, or negative; **child's perception of the donor** was rated as important, or showed little interest in him.

#### Mothers' feelings about the disclosure decision and the donor

**Current concerns over disclosure decision** was rated as none/minor concerns, or definite concerns; **feelings about identifiable donor** as positive, or neutral/ambivalent/negative; **importance of child's knowledge of genetic origins** as not/somewhat important, or extremely important.

To establish inter-rater reliability, 20 interviews were coded by a second rater. Spearman's rhos ranged from 0.82 to 0.94. Comparisons between the solo mother and two-parent families were conducted using t-tests and Chi-square tests of significance.

## Results

### The disclosure decision

Seventeen (54.8%) solo mothers had told their child about their donor conception, three (9.7%) had partially disclosed and 11 (35.5%) had not disclosed any details of the child's conception. Of the 14 (45.2%) solo mothers who had not disclosed or had only partially disclosed, all but one (92.9%) were intending to tell the child about their donor conception in the future and the remaining mother (7.1%) was uncertain. Seventeen (36.2%) of the partnered mothers had disclosed, four (8.5%) had partially disclosed and 26 (55.3%) had not disclosed. Of the 30 partnered mothers who had not disclosed or had only partially disclosed, 16 (53.3%) intended to tell the child in the future whereas six (20.0%) were uncertain and eight (26.7%) planned not to tell. As shown in Table 2, there was no difference in mothers' disclosure status by family type. A significant difference, however, was found in their disclosure intention, χ^2^(3) = 8.97, *P* < 0.05, reflecting a higher proportion of partnered mothers who were either uncertain or planned not to tell their child about their donor conception compared with solo mothers, none of whom had decided against disclosure.

Most (30 [96.8%]) solo mothers reported no or minor difficulties in their decision-making about disclosure, with only one (3.2%) experiencing definite difficulties. Whereas 39 (83.0%) partnered mothers similarly reported no or minor difficulties, eight (17.0%) reported definite difficulties (Table 2).

### The disclosure process

Eleven (64.7%) solo mothers had used a story book about sperm donation to first tell their child about their conception. A significant difference was found in the use of story books by family type (χ^2^[1] = 7.28, *P* < 0.05), with notably all (17 [100%]) partnered mothers who had disclosed having done so using such a book.

In families in which parents had disclosed, four (23.5%) solo mothers reported that these conversations were initiated equally by the mother and the child, five (29.4%) as predominantly by the mother and eight (47.1%) as predominantly by the child. By comparison, in two-parent disclosed families, two (11.8%) mothers reported that the child initiated these conversations, two (11.8%) that the child and parents did so equally and 13 (76.5%) that it was predominantly the parents. A significant difference was found between family types in who initiated these discussions (χ^2^[2] = 7.82, *P* < 0.05), with the child being more likely to bring up the topic of their donor conception in solo mother than in two-parent families. No difference between family types, however, was found in the frequency of conversations, with more than 61% of solo mothers and 67% of partnered mothers reporting that conversations about donor conception occurred at least once every 3 months. The large majority of both solo mothers (16 [94.1%]) and partnered mothers (16 [94.1%]) reported either no problems or feeling only mildly uncomfortable or distressed when having these discussions, with no difference in level of discomfort according to family type.

Most solo mothers who had disclosed either reported their child to have neutral or mixed feelings about being donor conceived (9 [52.9%]) or were unsure of their child's feelings (6 [35.3%]); whereas only two (11.8%) described positive feelings, none reported their child to have predominantly negative feelings. The partnered mothers similarly reported their child either to show neutral or mixed feelings (8 [47.1%]) or the mothers were unsure of their child's feelings (8 [47.1%]). None described positive feelings and only one (6%) reported their child to exhibit negative feelings. No difference was found in children's feelings about being donor conceived between family types.

According to mothers' reports of their children's perceptions of their donor, a minority of children of solo mothers perceived their donor as important (4 [23.5%]), with most (13 [76.5%]) displaying little interest in him. Only two (11.8%) children of partnered mothers viewed their donor as important, whereas the remaining 15 (88.2%) children showed little interest in him. No difference was found between the solo mother and partnered mother families in mothers' reports of children's perceptions of their donor.

### Mothers' feelings about the disclosure decision and the donor

Only one solo mother (3.2%) had definite concerns about her disclosure decision. This mother had not disclosed. The remaining solo mothers (30 [96.8%]) reported either no concerns or minor concerns only. No difference was found in the level of current concern about their disclosure decision between the solo and partnered mothers. Most solo mothers (26 [83.9%]) were positive about using an identifiable donor. Although this was also true of partnered mothers (28 [59.6%]), a significantly higher proportion of partnered than solo mothers had neutral, ambivalent or negative feelings (χ^2^ [1] = 5.18, *P* < 0.05). Furthermore, most solo mothers (23 [74.2%]) believed children's knowledge of their genetic origins to be extremely important compared with 46.8% of partnered mothers. The solo and partnered mothers differed significantly in the level of importance they placed on this (χ^2^ [1] = 5.74, *P* < 0.05), with a higher proportion of solo than partnered mothers believing children's knowledge of their genetic origins to be extremely important (Table 3).

### Disclosure and non-disclosure: the role of family type

A close examination of mothers' descriptions of the disclosure decision further revealed the role that family type played. For solo mothers, telling or not telling their child about their donor conception was intimately tied up with the issue of father absence. For example, disclosure could be prompted by a child's questions or realizations about the absence of a father in the home, as described by a mother of a 7-year-old child:

I just don't feel the need to lie to him about it… It's always been done on a real, as he's asked. I've not bombarded him with information, it's when he comes to me. I think the first time it ever actually came up in a conversation he was about 3 and a half, maybe 3, and a friend of his … asked him ‘where is your dad, have you got a dad?’. And I could see him thinking ‘hold on a second, I don’t know’. (solo mother)

Similarly, non-disclosure could necessitate the deflection of children's questions about the absence of a father. As one solo mother with a 4-year old described:

[Child] is small now so it's easy to just deflect and talk about something else, but it will get to a point where it's going to be harder…[Child] has asked once or twice, ‘where's my dad?’, and I've just sort of said ‘he's not around’… I'll have to answer the questions at some point. (solo mother)

It was also evident, however, that not all children had directly asked these questions, even those who were older, as exemplified by the following mother of an 8-year old's experience:

[Child] knows that she hasn't got a dad. And that's it at the moment…I think as soon as she starts to ask questions, and she realises how babies are born, then the next, the natural next question is, “How was I born? If I didn't have a dad, how was I born?” So she needs to know, you know, how she was born, and how she was conceived. (solo mother)

For solo mothers, disclosure decision-making was closely connected to the issue of father absence; however, for mothers in two-parent families, it brought up themes relating to the presence of the child's father in the home. This difference is illustrated by the following solo and partnered mothers' conception stories for their child, for whom disclosure entailed explaining the absence of a ‘daddy*’* to provide sperm and the absence of sperm from ‘daddy*’* respectively:

So you need to have a mummy part and a daddy part. And mummy had the mummy part and knew that she wanted to have a baby, but she didn't have a daddy to give her the daddy part. (solo mother)

It was very simple, she was only, she was 3 at the time so we just talked about needing a sperm and an egg, one from mummy and one from daddy, and that daddy didn't have any sperm so we needed to borrow one. (partnered mother)

Similarly, the significance of father presence featured in partnered mothers' narratives of non-disclosure, with the presence of the child's father underpinning some mothers' explanations for not telling their child about their donor origins:

I don't think it's important because [the donor conception] just was the process of being made, and actually her father is [father]…I wouldn't want her to feel that [father] wasn't her Dad. (partnered mother)

Having an identifiable donor added a further layer of complexity about whether, when and how mothers give information about the potential accessibility of the donor to their child. The disclosure of the identifiable nature of the donor is illustrated below:

I sometimes slip in as we're reading, it's not in the book but I sometimes do say, and if you want to, sweetie, when you're older you can, you can get in touch with him [the donor]. (solo mother)

During the course of the interviews, it became apparent that not all mothers had told, or planned to tell, their child that it may be possible to contact the donor in the future. To explore this issue further, an additional question was included for partnered mothers in the disclosed group asking whether they had informed their child that the donor was identifiable, and less than half (7 [41.2%]) responded that they had.

## Discussion

Despite the use of identifiable donors, only around one-half of solo mothers and one-third of partnered mothers had told their child about their donor conception, with no significant difference in disclosure rates by family type. A difference, however, was found between family types in intention to disclose, reflecting a higher proportion of solo mothers than partnered mothers intending to disclose but not yet having done so. As well as challenging the common assumption that children in solo mother families are inevitably told about their donor conception during their preschool years to explain the absence of a father, the low rate of disclosure among 4–8-year-old children in both family types has wider implications given the potential impact of a child's age on their response to finding out about their donor origins, with first disclosure in early childhood generally considered to be optimal (Nuffield Council on Bioethics, 2013). Although most of the non-disclosing solo mothers and one-third of the non-disclosing partnered mothers planned to tell their child about their donor conception in the future, previous longitudinal research has shown that the intention to disclose does not always translate into actual disclosure (Readings et al., 2011).

As expected from previous research (Graham, 2014), most of these solo mothers with identifiable donors expressed a preference for this type of donation. Although this was also true of partnered mothers, a higher proportion of mothers in two-parent families expressed neutral, ambivalent or negative feelings about open-identity donation. Indeed, the rate of full disclosure among partnered mothers (36.2%) was not substantially higher than that found in the latest comparable UK study of heterosexual couple families with anonymous donors which identified a 28% disclosure rate at age 7 years (Golombok et al., 2011). This suggests that identifiable donation has not so far had a major effect on rates of disclosure. The finding that less than one-half (46.8%) of the partnered mothers considered children's knowledge of their genetic origins to be extremely important compared with most (74.2%) of the solo mothers is in line with the lower disclosure intention rate in the two-parent families.

As reported in previous research on heterosexual couple families, this study found that children who had been told about their donor conception in their early years seemed to assimilate this information with a relatively neutral stance. This indicates that a ‘seed planting’ approach to disclosure by which children feel they have always known about their donor conception (MacDougall et al., 2007) is associated with more positive outcomes than later disclosure. Although family type was seen to influence mothers' experiences of disclosure, no differences between family types were identified in children's feelings about their donor conception or interest in their donor, with most children in both family types showing little interest in him. Both groups of mothers, but especially the partnered mothers, found story-books helpful in telling their children about their donor conception. Although conversations about donor conception seemed to occur at a similar frequency in both family types, it seemed that the children of solo mothers felt more comfortable in initiating these conversations suggesting greater openness in communication on this topic in solo mother families.

A limitation of studies of disclosure in donor conception families concerns their representativeness. It is possible that parents who do not agree to participate in research may be more inclined towards non-disclosure. To the extent that this is the case, the findings of the present study are likely to represent an over-estimate of disclosure rates in families formed using identifiable donors. A further limitation relates to the relatively small sample sizes and associated low levels of statistical power. Larger samples are required to establish whether significant effects were not detected due to sample size constraints. A further limitation is that the findings represent parents' disclosure experiences at one time point when their children are relatively young. Mothers' disclosure decisions may change over time.

The findings of this study have implications for the provision of counselling in relation to donor identification. In particular, it should not be assumed that parents using identifiable donors will necessarily tell their children about their genetic origins. Therefore, donor-conceived children may not be aware of their entitlement to request the identity of their donor at age 18 years. The findings point to the need for longitudinal research to establish not only if and when parents disclose donor conception to their children but also if and when they inform their children that they are legally entitled to discover the identity of their donor on reaching age 18 years.

## Figures and Tables

**Table 1:** Sociodemographic characteristics of sample by family type.

	Solo mothers		Partnered mothers			
	Mean	SD	Mean	SD	*F*	*P*
Age of mother (years)	43.71	3.36	38.83	3.86	33.04	<0.001
Age of child (months)	56.71	10.71	59.89	12.08		NS

	*n*	%	*n*	%	χ^2^	*P*
Child sex						
Male	14	45.2	27	57.4	1.13	NS
Female	17	54.8	20	42.6		
Siblings						
None	19	61.3	14	29.8	8.27	<0.05
One	10	32.3	23	48.9		
Two or more	2	6.5	10	21.3		
Mother's education						
Below university degree	10	32.3	22	46.8	1.69	NS
Undergraduate degree	11	35.5	14	29.8		
Postgraduate degree	10	32.3	11	23.4		
Mother's working status						
Not working	9	29.0	14	29.8	0.01	NS
Working	22	71.0	33	70.2		
Perceived financial difficulties						
None	27	87.1	40	85.1	0.11	NS
Minor	2	6.5	4	8.5		
Definite	2	6.5	3	6.4		

NS, not significant.

**Table 2:** Mothers' disclosure status, disclosure intention and difficulties in decision-making by family type.

	Solo mothers		Partnered mothers			
	*n*	%	*n*	%	χ^2^	*P*
Disclosure status						
Disclosed	17	54.8	17	36.2	3.07	NS
Partially disclosed	3	9.7	4	8.5		
Not disclosed	11	35.5	26	55.3		
Disclosure intention						
Disclosed	17	54.8	17	36.2	8.97	<0.005
Plans to tell	13	41.9	16	34.0		
Uncertain	1	3.2	6	12.8		
Plans not to tell	0	0	8	17.0		
Decision-making						
None or minor difficulties	30	96.8	39	83.0	3.48	NS
Definite difficulties	1	3.2	8	17.0		

NS, non-significant.

**Table 3:** Mothers' feelings about the disclosure decision and the donor by family type.

	Solo mothers		Partnered mothers			
	*n*	%	*n*	%	χ^2^	*P*
Current concerns over disclosure decision						
None or minor concerns	30	96.8	46	97.9	0.09	NS
Definite concerns	1	3.2	1	2.1		
Feelings about identifiable donor						
Positive	26	83.9	28	59.6	5.18	<0.05
Neutral, ambivalent or negative	5	16.1	19	40.4		
Importance of child's knowledge of genetic origins						
Not or somewhat important	8	25.8	25	53.2	5.74	<0.05
Extremely important	23	74.2	22	46.8		

NS, non-significant.
